# Self-Organized Structuring of Recurrent Neuronal Networks for Reliable Information Transmission

**DOI:** 10.3390/biology10070577

**Published:** 2021-06-24

**Authors:** Daniel Miner, Florentin Wörgötter, Christian Tetzlaff, Michael Fauth

**Affiliations:** Bernstein Center for Computational Neuroscience, Third Institute of Physics, Georg-August University, Friedrich Hund Platz 1, 37077 Göttingen, Germany; danielcarlminer@gmail.com (D.M.); worgott@gwdg.de (F.W.); tetzlaff@phys.uni-goettingen.de (C.T.)

**Keywords:** self-organization, synaptic plasticity, information transfer

## Abstract

**Simple Summary:**

Information processing in the brain takes places at multiple stages, each of which is a local network of neurons. The long-range connections between these network stages are sparse and do not change over time. Thus, within each stage information arrives at a sparse subset of input neurons and must be routed to a sparse subset of output neurons. In this theoretical work, we investigate how networks achieve this routing in a self-organized manner without losing information. We show that biologically inspired self-organization entails that input information is distributed to all neurons in the network by strengthening many synapses in the local networks. Thus, after successful self-organization, input information can be read out and decoded from a small number of outputs. We also show that this way of self-organization can still be more energy efficient than creating more long-range in- and output connections.

**Abstract:**

Our brains process information using a layered hierarchical network architecture, with abundant connections within each layer and sparse long-range connections between layers. As these long-range connections are mostly unchanged after development, each layer has to locally self-organize in response to new inputs to enable information routing between the sparse in- and output connections. Here we demonstrate that this can be achieved by a well-established model of cortical self-organization based on a well-orchestrated interplay between several plasticity processes. After this self-organization, stimuli conveyed by sparse inputs can be rapidly read out from a layer using only very few long-range connections. To achieve this information routing, the neurons that are stimulated form feed-forward projections into the unstimulated parts of the same layer and get more neurons to represent the stimulus. Hereby, the plasticity processes ensure that each neuron only receives projections from and responds to only one stimulus such that the network is partitioned into parts with different preferred stimuli. Along this line, we show that the relation between the network activity and connectivity self-organizes into a biologically plausible regime. Finally, we argue how the emerging connectivity may minimize the metabolic cost for maintaining a network structure that rapidly transmits stimulus information despite sparse input and output connectivity.

## 1. Introduction

The brain’s visual system continuously transmits perceived information along a hierarchical topology. At each layer of this topology, predominantly short-range connections form a recurrent neuronal network while long-range connections convey inputs from the previous layer and send outputs to the subsequent layer. Hereby, the long-range connections are sparse, meaning that a neuron in one layer has a rather low probability to be connected to a neuron in the subsequent (target) layer. Sparsity across layers leads directly to the problem of routing the information from an input neuron via the recurrent network of the layer to a specific output neuron.

The foundation of the brain’s topology is determined during development and the coarse of the morphology of axons and dendrites remains rather rigid afterwards [1]. The chemo-affinity hypothesis formulates that during development gradients of chemical signals [2] being sensed by axonal surface molecules [3,4] guide the axons towards their destination area. Hereby the molecular composition defines a “molecular code” to specify the target neuron. However, the low number of guidance molecules [5] and the stochasticity of the involved processes [6] impede the clearness of the code. By contrast, anatomical studies imply a rather unspecific assignment of neurons [7,8,9]. Hence, in the worst case, which we consider in this study, the sparse in- and output connections of a layer could be unstructured and assigned randomly to different neurons.

After the initial developmental phase, the brain also has the ability to adapt to sensory experiences. For this, neuronal networks modify the properties of their neurons and synapses by means of local activity-dependent plasticity processes. Most prominently, synaptic plasticity adapts the transmission efficacy or weights of the established connections or synapses [10,11,12,13,14,15,16,17]. On the other hand, homeostatic processes such as synaptic scaling [16,18,19] and intrinsic plasticity [20,21] regulate synaptic weights and the excitability of neurons, respectively, such that the neuronal activity remains within a desired regime. A series of theoretical studies shows that the interplay of these processes leads to the self-organized formation of memory representations of observed input stimuli [19,22] and their correlations [23,24], while matching and explaining the experimentally observed connectivity on the microcircuit level [25,26]. These studies focused predominantly on investigating the neuronal and synaptic dynamics within the recurrent network forming one hierarchical layer neglecting its embedding in the hierarchical topology discussed before.

In this study, we investigate whether the interplay of activity-dependent plasticity mechanisms self-organizes a recurrent neuronal network to support the decoding of input stimuli by subsequent network layers on the condition that all connections are sparse, random and their source and target neurons are predetermined. Utilizing a mathematical neuronal network model of the visual cortex [23] matching a multitude of experimental data [25,26], in the first step, we show that the self-organizing principles lead to an optimal decoding performance despite sparse, random read-out connections. In the second step, we identify the effect of the self-organization on the neuronal activity and the information storage of each neuron in the recurrent network resulting in the decoding performance. Moreover, we investigate the underlying adaptation of the synaptic weight structure and compare it to experimental data. Finally, we provide an analysis suggesting that the resulting network architecture could be designed for low metabolic maintenance costs.

## 2. Methods and Materials

### 2.1. Model Network

Simulations are implemented in the Brian 2 simulator platform [27] and follow implementations of self-organizing spiking networks from earlier work [25,28].

**Figure 1:**
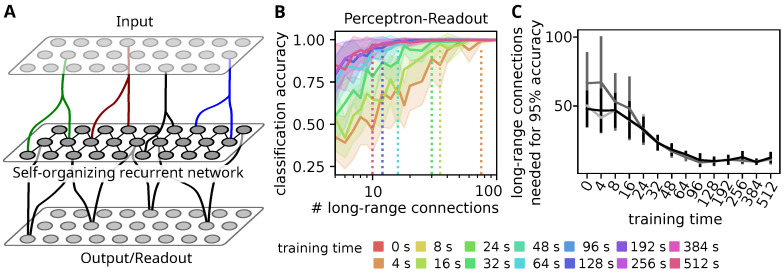
Self-organization of recurrent networks allows for efficient information transmission by sparse fixed in- and output-projections. (**A**) Situation considered in this study: (Sensory) information is processed in a hierarchy of recurrent networks. Connections between layer of the hierarchy are costly, such that each layer receives sparse input and transmits outputs only from a subset of its cells via long-range output projections. As those require long-range axons, they are fixed and cannot be reorganized in response to stimulation. (**B**) Accuracy of a perceptron classifying the presented stimulus from the activity of a random sub-set of neurons in the self-organizing network. Different colors indicate training time (0 s for network without self-organization). Dashed lines indicate the minimum number of samples needed to achieve 95% classification accuracy. Initially around 100 neurons are needed but after around 100 s of self-organization, 10–15 neurons are sufficient to decode the stimulus identity. Transparent regions mark standard deviations of classification accuracy for a stratified 5-fold cross-validation and 6 different choices of readout connections. (**C**) Number of long-range connections needed to achieve at least 95% classification accuracy at different training times. Perceptron classifier (black curve) and non-neuronal classifiers such as 3-nearest-neighbor (light grey) or a (linear) support-vector machine (dark grey) arrive at similar results. Data points are mean and standard deviation of the last number of connections with more than 95% accuracy from 40 decreasing sequences.

The self-organizing recurrent network model (Figure 1A) consists of a population of $n_{E}=1000$ excitatory neurons with absolute refractory periods of $\tau_{E}^{\mathrm{refrac}}=10\;\mathrm{ms}$ and $n_{I}=200$ inhibitory neurons with absolute refractory periods of $\tau_{I}^{\mathrm{refrac}}=2\;\mathrm{ms}$.

We recurrently connect the excitatory neurons (without self-connections) and interconnect the inhibitory and excitatory pool (in both directions) with random sparse connections with probability $p_{\mathrm{connect}}=0.04$. Recurrent inhibitory connections are neglected. Recurrent excitatory connections are given an initial strength of w=0.5nS, and all other connections are given an initial strength of w=1.0nS.

#### 2.1.1. Neuron Model

We use conductance-based leaky integrate-and-fire neurons [29], with membrane voltages evolving according to:

(1) $$\frac{dv_{j}}{dt}=\frac{g_{\mathrm{leak}}\left(v_{\mathrm{rest}}-v_{j}\right)+g_{\mathrm{ampa},j}\left(v_{\mathrm{ampa}}-v_{j}\right)+g_{\mathrm{gaba},j}\left(v_{\mathrm{gaba}}-v_{j}\right)}{c_{\mathrm{membrane}}}+\frac{\sigma_{\mathrm{noise}}\xi }{\sqrt{\tau_{\mathrm{membrane}}}},$$

and synaptic conductances evolving according to

(2) $$\frac{dg_{\mathrm{ampa},j}}{dt}=-\frac{g_{\mathrm{ampa},j}}{\tau_{\mathrm{ampa}}}\;\mathrm{and}\;\frac{dg_{\mathrm{gaba},j}}{dt}=-\frac{g_{\mathrm{gaba},j}}{\tau_{\mathrm{gaba}}}.$$

Here, $v_{j}$ is the membrane voltage of neuron *j*, $g_{\mathrm{leak}}$ is the leak conductance, $v_{\mathrm{rest}}$ is the resting potential, $c_{\mathrm{membrane}}$ is the membrane capacitance, $\tau_{x}$ is the time constant for feature x, $g_{\left[\mathrm{ampa},\;\mathrm{gaba}\right],j}$ is the conductance for each neurotransmitter type, $v_{\left[\mathrm{ampa},\;\mathrm{gaba}\right]}$ is the reversal potential for each neurotransmitter type. ξ is an Ornstein-Uhlenbeck noise generator, and $\sigma_{\mathrm{noise}}$ is the noise variance. Please refer to Table 1 for the chosen parameter values.

#### 2.1.2. Neural Adaptation

The firing threshold of neuron *j*, $v_{\mathrm{threshold}}^{j}$, is adaptive (see [30] for a review) and follows:

(3) $$\frac{dv_{\mathrm{threshold}}^{j}}{dt}=-\eta_{\mathrm{decay}}^{\mathrm{ip}}$$

with adaptation rate $\eta_{\mathrm{decay}}^{\mathrm{ip}}$. When $v_{j}>v_{\mathrm{threshold}}^{j}$, we reset the potential $v_{\mathrm{rest}}\to v_{j}$ and set $v_{\mathrm{threshold}}^{j}+\eta_{\mathrm{spike}}^{\mathrm{ip}}\to v_{\mathrm{threshold}}^{j}$, as well as $g_{\mathrm{ampa},i}+w_{ij}\to g_{\mathrm{ampa},i}$ or $g_{\mathrm{gaba},i}-w_{ij}\to g_{\mathrm{gaba},i}$, respectively. Here, $\eta_{\mathrm{spike}}^{\mathrm{ip}}$ is the adaptation increment for the intrinsic firing threshold plasticity, and $w_{ij}$ is the strength of the synaptic connection from presynaptic neuron *j* to postsynaptic neuron *i*.

#### 2.1.3. Synaptic Plasticity and Normalization

The synaptic weights $w_{ij}$ between two excitatory neurons are modified by spike timing-dependent plasticity (STDP) [14,15,31,32], which changes them according to the temporal difference between adjacent pre- and postsynaptic spikes Δt:

(4) $$\Delta w_{ij}=\left\{\begin{array}{cc} A_{+}\exp\left(-\Delta t/\tau_{+}\right) & \Delta t>0,\\ A_{-}\exp\left(\Delta t/\tau_{-}\right) & \;\;\Delta t<0,\\ 0 & \;\;\Delta t=0. \end{array}\right.$$

Here, $A_{+/-}$ is the plasticity amplitude, $\tau_{+/-}$ is the decay time constant, and + and − signify potentiation and depression, respectively.

After each STDP-induced weight change, we also implement a biologically inspired synaptic normalization mechanism [18,33,34] modeled by

(5) $$\begin{array}{c} W_{i}\frac{W_{\mathrm{total}}}{\left|\right|W_{i}{\left|\right|}_{1}}\to W_{i} \end{array}$$

where $W_{i}$ is the vector of incoming weights to neuron *i*, $\left|\right|W_{i}{\left|\right|}_{1}$ its L1-norm, and $W_{\mathrm{total}}$ the target value for the total incoming weight.

### 2.2. Training and Testing Paradigm

Our goal is to study how network self-organization shapes the transmission of information conveyed by stimuli. To this end we simulate the self-organization of a recurrent network which is exposed to five different stimuli for varying durations and then assess the network properties. All simulations are conducted in four phases:

(1) **Warm-up:** The network is simulated without input for a period of 50 s to allow all dynamical variables to converge to an equilibrium distribution.
(2) **Training:** In the following, five non-overlapping stimulation groups of 40 excitatory neurons are driven through strong connections (20 nS) from a group-specific Poisson spike source firing at 50 Hz when activated. Every 200 ms another source is activated for 100 ms.
(3) **Relaxation:** Afterwards, plasticity and inputs are turned off and the homeostatic mechanisms are allowed to re-equilibrate for 50 s.
(4) **Testing:** The network is presented with recall cues which consist of one precisely timed input spike to all neurons in one stimulation-group conveyed through a strong (20 nS) connection. To allow for sufficient network relaxation, there is only one recall stimulus every 500 ms for 100 s.

To assess the influence of the training phase duration, we conduct new simulations with the respective training phase duration every time.

### 2.3. Evaluation Measures

#### 2.3.1. Classification Accuracy

To assess whether information about the stimulus is present in the activity of a certain brain area or, as considered here, in the activity of a subset of cells (with output projection) a decoding analysis can be performed (for a recent review see [35]). Hereby, a decoder–that is a mapping from the neural activities to the stimulus–is derived and it probability to predict the right stimulus (accuracy) is evaluated. Our focus in this study lies on the question if information can be decoded by a subsequent network rather than how this is achieved by a neuronal system. Therefore, we use standard classification algorithms without asking whether they can be neuronally implemented. The latter question is subject to intensive research and out of the scope for this study.

As the most biologically inspired algorithm, we employ a perceptron classifier using the implementation from the scikit-learn-framework [36]. This algorithm uses a single readout neuron for each stimulus-class, which is trained to display high activity when the respective stimulus has been presented and low activity for all others. The prediction of the classifier amounts to the stimulus corresponding to the readout neuron with maximal activity (one-vs-all multi-class classification).

As a comparison we also use well-established machine learning algorithms: a support vector machine with linear kernels and a k-nearest neighbor classifier (with k = 3), both also using the standardized implementation from the scikit-learn-framework.

To determine, if a certain number of long-range projections allows sufficient decoding, we repeatedly randomly select the given number of neurons, and train the above classifiers on their immediate spiking response to different stimuli (2.5 ms in 0.5 ms bins). To estimate the classification accuracy, we use only 80% of the responses for training and test the prediction on the remaining 20%. This process is repeated five times using a stratified cross-validation strategy.

#### 2.3.2. Analytical Approximation of the Decoding Accuracy Depending on Response Probabilities

To approach the decoding accuracy analytically, we first consider the situation where neurons respond to one preferred stimulus with probability $p_{on}$ and to all other stimuli with $p_{off}$, which approximately corresponds to the response behavior of our recurrent networks after self-organization. We assume that our decoder receives input from an equal number $n_{con}$ of neurons tuned to each of the $n_{stim}$ stimuli and the firing of these neurons is independent. The output of our decoder is assumed to be the stimulus corresponding to the population of inputs with the same tuning that spiked most often. In that case, the probability for the decoder to yield the right output is the probability that the neurons tuned to the presented stimulus fire more than any other group of neurons. Using Bernoulli distributions, this probability can be evaluated as:

Pdecode(pon,poff)=∑k=1nconnconkponk(1−pon)ncon−k∑k˜=0k−1nconk˜poffk(1−poff)ncon−knstim−1.

**Figure 2:**
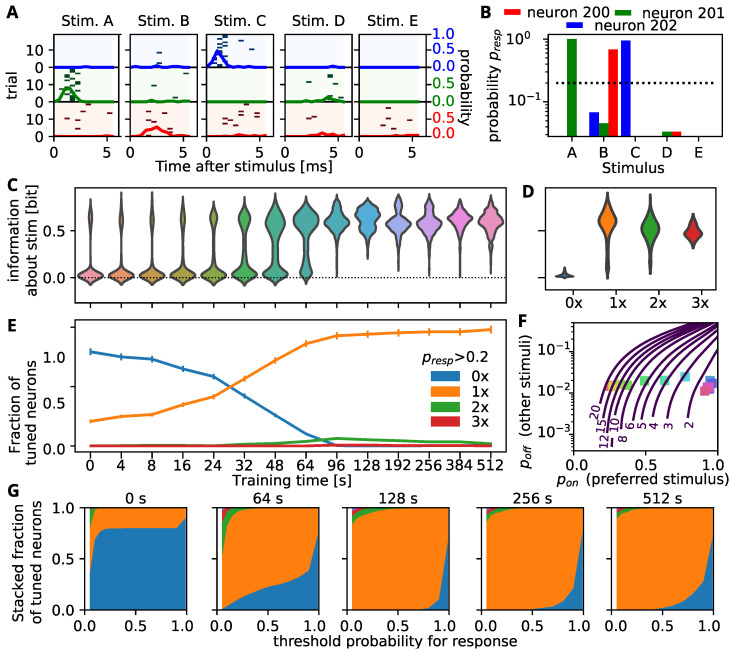
Self-organization tunes all neurons reliably to a single stimulus allowing for readout with less connections. (**A**) Stimulus triggered responses (spike rasters for 10 trials and mean response) to the five possible stimuli for three (unstimulated) cells (colors) after 48s of training. At this stage, clear preferences for single stimuli are visible. (**B**) Spiking probability withing the fist 2.5ms after the stimulus for the three example cells. If the probability for a stimulus exceeds the threshold (dashed line), the cell is considered to be tuned to that stimulus. (**C**) Distributions of mutual information between stimulus and single neuron activity (up to 2.5 ms after the stimulus) at different training times. (color coded as in Figure 1B). (**D**) Distribution of the mutual information for neurons that respond to the same number of stimuli (with more than 20% probability) indicated on the x-axis. Neurons responding to one or two stimuli convey maximal information. Color code for number of tunings as in panel E. (**E**) Evolution of the the fraction of neurons that responds to n = 0, 1, 2, 3 or 4 stimuli. (**F**) Response probabilities for preferred stimulus ($p_{on}$, x-axis) and all other stimuli ($p_{off}$, y-axis) determine the number of readout connections needed to decode the stimulus. Blue curves mark the boundary between combinations of response probabilities for which the stimulus can be decoded from the indicated number of neurons per stimulus with a 95% accuracy. Colored dots mark the mean response probabilities observed in simulations. Color code as in Figure 1B. (**G**) Fraction of neurons responsive to n-stimuli depending on the response threshold at different times during network training (indicated in title). Color code for number of tunings as in panel E. After training most neurons respond to a single stimulus.

For a given number of stimuli, we evaluated these probabilities for all combinations of $p_{on}$ and $p_{off}$ and obtained the 95% isolines for a varying number of connections per stimulus $n_{con}$. In Figure 2F the resulting isolines are shown for five stimuli corresponding to the situation in the simulations.

### 2.4. Number of Long-Range Connections Needed to Decode Partly Tuned Networks

The above analysis assumes that each long-range output connection emerges from a tuned neuron and that there are approximately equally distributed between the stimuli. Next, we investigate how the number of outputs needed to decode the stimulus scales if these assumptions do not hold. To this end, we consider a network where a fraction *f* of the neurons are tuned to one of $n_{stim}$ stimuli and respond to it with $p_{on}=1$ while not responding to other stimuli ($p_{off}=0$). The remaining fraction 1−f of neurons is untuned. We further assume that the preferred stimuli are randomly chosen from the $n_{stim}$ stimuli and every stimulus occurs with equal probability $f/n_{stim}$. The stimulus identity can be reliably read out from the network, if at least $n_{stim}-1$ differently tuned neurons can be identified by the read outs. We determine the probability to observe at least $n_{stim}-1$ differently tuned neurons after *k* draws following [37]:

p(k)=∑l=0kklfk−l(1−f)l∑m=1nstim∑s=0min(nstim−m,1)nstimm(−1)nstim−m−s(nstim−m)smnstimk−l.

To determine the minimal number of needed long-range connections to decode all stimuli with 95% certainty, we steadily increase the number of draws *k* until p(k)>0.95. An analytic approximation of the number of needed long-range connections can be found in the Appendix A.

#### 2.4.1. Mutual Information

Decoding analyses can identify whether information on the stimulus is present in neuronal activities, but it is difficult to interpret how it is encoded and how individual neurons contribute to the decodability, partly because this depends on the decoder-model [35]. As a model-free measure of the information that the activity of each single neuron carries about the stimulus we use mutual information [38,39,40]. This measure tells us how much information we gain about one random variable–here, the stimulus–by observing the other–here, the neuronal responses. Specifically, we calculate the mutual information between single neuron responses (spike or no spike) and the stimulus by subtracting the stimulus conditioned entropy of the responses H(response|stimulus) from the unconditioned entropy of the responses H(response):

MI(response,stimulus)=H(response)−H(response|stimulus).

To determine theses entropies, we use the probability $p_{i}$ that a neuron fires within the first 2.5 ms after presentation of stimulus i∈{A,B,C,D,E}:

$$MI\left(response,stimulus\right)=H_{2}\left(\frac{1}{5}\sum_{i\in \{A,B,C,D,E\}}p_{i}\right)-\sum_{i\in \{A,B,C,D,E\}}H_{2}\left(p_{i}\right)$$

where $H_{2}\left(p\right)=-p\cdot log_{2}\left(p\right)-\left(1-p\right)\cdot log_{2}\left(1-p\right)$.

Note that, as the neurons response is binary (spike or no spike, time is not taken into account), this mutual information is maximally 1 bit, when the neuron spikes in 50% of the cases. However, if the cell only responds to one stimulus with 100% probability–that is in 20% of the cases if the stimuli occur equally often–the value would be $-0.2log_{2}\left(0.2\right)-0=0.46\;\mathrm{bit}$.

#### 2.4.2. Correlation Dependent Densities

To assess whether network self-organization entails strong connectivity between correlated neurons as observed in [41], we perform a similar analysis as in these experiments: For each existing synapse, we determined the correlation of a pre- and postsynaptic neuron pair using the Pearson correlation coefficient of the stimulus responses of the respective neurons. Hereby, we restricted the responses for each trial to the first 2.5 ms after the stimulus presentation. We then sorted the synapses according to these correlation coefficients and evaluated the cumulative sum of synaptic weights as well as the number of synapses for increasing correlations, similar to the analysis conducted in [41].

## 3. Results

### 3.1. Self-Organization Improves Stimulus Decoding through Sparse Readouts

As explained above, networks within the cortical hierarchy need to relay information on incoming sensory stimulation from a subset of neurons that receive sparse input connections from the previous layer of the hierarchy to another subset of neurons from which sparse output connections project to the next layer of the hierarchy (Figure 1A). Here we investigate how recurrent neuronal networks can achieve such information routing through self-organization. To this end, we investigate the stimulation, reorganization and readout of self-organizing recurrent networks (SORN) comprising 1200 conductance-based leaky integrate-and-fire neurons equipped with intrinsic plasticity, spike-timing-dependent plasticity and synaptic normalization [23,25].

As a first step, we test whether self-organization improves the described information routing, by testing how well a presented stimulus can be decoded from the activities of a subset of output neurons at different stages of the self-organization. For this, the network is presented with five stimuli, which are modeled by increased rates in one of five input populations, whose activity is conveyed to the network by sparse input connections. The network is then allowed to self-organize in response to these stimuli for a specified training time.

We then test, whether the stimulus can be identified from a sparse readout that only sees the activity of a small subset of neurons in the recurrent network (Figure 1A). For this, we track the spiking activity relative to the stimulus presentation time and try to decode the stimulus from a sub-set of the recorded neuron activities using multiple classifiers. To this end, we repeatedly select a subset of neurons and divide their responses into a training set, on which a decoder is trained, and a test set on which its classification accuracy is evaluated. At the beginning of the training the decoding accuracy rises only slowly with the number of neurons and a good accuracy above 95% is only reached with around 100 neurons (Figure 1B,C). After around 100 s of training the accuracy rises much faster such that only 10–15 neurons are needed to decode the stimulus with high accuracy (Figure 1B). This result is preserved when other classifiers are used (Figure 1C).

Thus, we conclude that self-organization improves the readout of stimulus information by sparse randomly distributed long-range connections to other layers or brain areas.

### 3.2. Self-Organization Distributes Information by Tuning All Neurons to a Single Stimulus

In the next step, we aim to identify the features of self-organized neuronal code in the recurrent network that allows for the above described improved decoding as training proceeds. Most prominently, we find that, after a short training interval, also the cells that receive no stimulation exhibit a rapid response, mostly to one of the stimuli (Figure 2A). However, as the actual time of the spike is slightly jittered from trial to trial (Figure 2A), we also tracked the cumulative probability that a cell spikes within the first 2.5 ms after the stimulus presentation. As expected from the spike-rasters, these distribution exhibit clear peaks for one of the stimuli and only small probabilities to fire for one of the other stimuli (Figure 2B).

We then set a threshold for the stimulus-dependent firing probability (20%, dashed line in Figure 2B) above which a cell is considered as tuned to the respective stimulus and evaluated how many cells are tuned to one, two or more stimuli at different training times. While initially most cells are tuned to no stimulus (Figure 2E, blue curve) and only the 200 cells that are stimulated are tuned to one stimulus (orange curve), the number of cells that are tuned to no stimulus decreases continuously over the first 100 s of training, such that at the end, every cell responds to external stimuli. Interestingly, nearly all of the cells become tuned to only one of the stimuli (orange curve), while double or triple tunings remain sparse (green/red curves).

We wondered how this strong preference for one of the stimuli influences the information that the activity of a single cell carries about the stimulus. Thus, we evaluated the mutual information between the stimulus and the activity of individual cells and tracked the distribution of this mutual information over time (Figure 2C; thickness of the shape signifies the relative frequency of mutual information value indicated at y-axis at the training time indicated at the x-axis). Initially the activity of only a few cells–presumably the stimulated ones–carries information about the stimulus, while the activity of all other cells is uninformative. As the number of cells that are tuned to at least one stimulus rises, the probability mass shifts towards higher mutual information values until ultimately the activity of each individual cell carries around 0.5 bit of information about the stimulus (training times larger than 96 s). Note, while in principle the spiking probability could carry up to 1bit of information, a perfectly tuned cell responding to one stimulus with 100% probability and remaining silent for the rest of the stimuli has a mutual information value of 0.46bit. This indicates that self-organization does not necessarily lead to a optimal representation of information in the spiking activity, but to one that can be easily read out (see below). Yet, when the mutual information distributions are calculated separately for cells that are tuned to different numbers of stimuli (Figure 2D), we find that cells with a tuning for one stimulus actually yield slightly higher information content than cells with multiple tunings. Hence, for the network we consider here, the final state with a single tuning for each cell might be the best choice.

The number of tunings, however, could strongly depend on the threshold for the firing probability that is used. Therefore, we varied the threshold for tuning and evaluated the fraction of tuned cells at different training durations (indicated in panel title, Figure 2G). The tendency to acquire a single tuning (orange) is observed over a large range of thresholds. Tunings to two or more stimuli are mostly observed for very small thresholds during the beginning of training (Figure 2G). At very high thresholds, the number of untuned neurons (blue) first shrinks and then rises again over time, which can be attributed to intrinsic plasticity which prevents the neurons from being overly active and responding to each stimulus presentation with 100% probability.

### 3.3. Decodability Improves through Increasing the Response to Preferred Stimulus

In summary, the above results indicate that the majority of neurons in the network responds to a single stimulus, but with a probability below 1. We use this simplified view to gain an analytical insight, how many long-range connections would be needed to read out from such a trained network and decode the stimulus with a given accuracy. For this we consider the situation, where each neuron is tuned to one preferred stimulus and responds to it with probability $p_{on}$, whereas it responds to all other stimuli with probability $p_{off}$. For a given number of long-range connections per stimulus, we can then calculate which combinations of $p_{on}$ and $p_{off}$ would allow us to discern the correct stimulus in 95% of the cases (by choosing the population with the maximal activity). In general, the higher the response probability to the preferred stimulus, the higher also the response probability for the other stimuli can be (Figure 2F, blue curves with number of long-range connections indicated). Using less connections however, will require higher response probabilities to the preferred stimulus or smaller ones to the not preferred stimulus. We then determined the average of these response probabilities for our simulated network in different training stages. The self-organization initially increases the average response probability to the preferred, but also to the not preferred stimuli (Figure 2F, square markers, color indicates training time). Later on, the response to the non-preferred stimuli is decreased again, most likely due to the homeostatic mechanisms that prevent excessive firing. Comparing the time-course of the response probabilities to the curves indicating the number of connections necessary for decoding reveals that self-organization transforms the network from a state where around 20 long-rage connections would be needed per stimulus, to a point where only one or two connections are necessary for a reliable readout, mainly by increasing the response probability to the preferred stimulus.

### 3.4. Tuning Spreads Due to Strong Feed-Forward Connections

The phenomenon that most cells tune to a single stimulus can be explained by the self-organization mechanisms in our network: The intrinsic plasticity mechanism adapts the firing threshold of the unstimulated cells to prevent them from being inactive. Hence, they eventually become very sensitive and can be triggered via random connections by the stimulated cells. The resulting correlated firing leads to a strengthening of those connections by STDP. As stronger weights also induce more correlated firing, this constitutes a positive feedback loop. However, synaptic normalization also introduces competition between input weights to a cell and, hence, a winner-take-all mechanism that ultimately only allows strong weights from the stimulated cells of a single stimulus.

**Figure 3:**
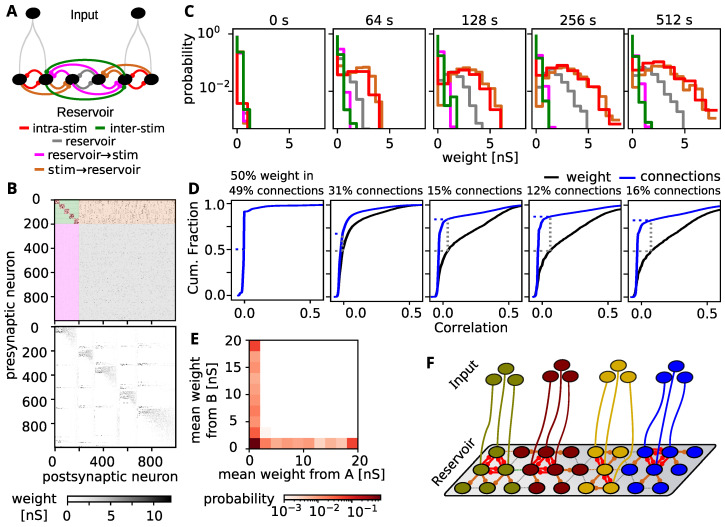
Self-organization of connectivity during training. (**A**) Connection-types emerging from the stimulation paradigm: We differentiate between intra-stimulus connections (red) between neurons stimulated by the the same input-group and inter-stimulus connections (green) between cells stimulated by different inputs. Furthermore there are connections from stimulated cells to the unstimulated cells (brown), between unstimulated cells (grey) and from the unstimulated to stimulated cells (magenta). (**B**, **top**) Excitatory synaptic weight matrix after 512 s training. Note the recurrent connection blocks between the stimulated neurons (0–199) and the feed-forward projections to the unstimulated neurons. Color code as defined in panel A. (**B**, **bottom**) Weight matrix with neurons resorted according to the stimulated neuron group from which they receive the maximum input and the size of that weight. Note, for better visibility, weight matrices have been sub-sampled using max-pooling with a stride of 4. (**C**) Histogram of the synaptic weights of the different connection types. Connections within and from stimulated groups form stronger weights. Also weights between unstimulated neurons increase. (**D**) Cumulative distribution of synaptic connections (blue) and synaptic weight (black) with connections sorted according to the correlation between pre- and postsynaptic signals. Similar as in experimental results from visual cortex (compare [42]), large fractions of the total synaptic weight stem from synapses between neuron pairs with high signal correlation. (**E**) Pairwise histogram of the mean incoming weights from the stimulated neuron groups receiving stimuli A and B for the same neuron. Neurons with large weights from one stimulated group do not have large weights from the other stimulated group. (**F**) Schematic drawing of the connectivity resulting by training. The network is partitioned in strongly connected sub-networks for each of the presented stimuli.

Evidence for this mechanism can be found in the time evolution of histograms of different weight types in our network (Figure 3A,C). First of all, neurons that receive the same stimulus exhibit correlated firing, such that the weights between them (intra-stimulus, red) are continuously growing. However, also the weights that project from the stimulated neurons to the unstimulated neurons (brown) grow at the same pace. The weights that project back to the stimulated neurons (reservoir → stim, pink), however, remain weak. This could arise from the asymmetric nature of the STDP rule, which senses that the stimulated neurons trigger the firing in the unstimulated ones, but not vice versa. Weights between unstimulated neurons (reservoir, grey) also grow, although a bit less than those from stimulated neurons. Finally, firing between neurons that receive different stimuli are uncorrelated and the weights between them remain low (inter-stimulus, green).

As a consequence, after training, we observe strong recurrent connections between the neurons receiving the same stimulus (Figure 3B top, indices 0–199, five groups with 40 neurons). Moreover, we observe strong connections from these neurons to the rest of the network (upper right block), but not back (lower left block) or between the unstimulated neurons (lower right block). When the neuron indices are rearranged according to the stimulated group from which they receive the strongest cumulative input weight and the size of that weight (Figure 3B bottom), the connectivity matrix exhibits a block diagonal structure, which is in line with the observation that each neuron tunes to one stimulus.

Furthermore, neurons that receive strong weights from one stimulated group, do not receive strong weights from another (Figure 3E), which likely emerges by the winner-take-all structure induced by synaptic normalization.

In summary, we see a spread out of tuned stimulus responses from the stimulated neurons to the whole unstimulated network (Figure 3F). Thus, the self-organizing dynamics determined by the interplay between STDP, synaptic normalization, and intrinsic plasticity allocates the maximum possible neuronal resources to represent the stimuli. Intuitively, this spread-out of the stimulus representation due to self-organization is beneficial for a sparse readout by a subsequent network layer or brain area. This is because all long-range readout connections–no matter how they are distributed–can be expected to sample from tuned neurons whose activity carries information about the stimulus (see above).

### 3.5. Comparison to Experimental Findings

We wondered whether the above-described interplay between neural activity and connectivity is biological plausible. To this end, we repeated an analysis from visual cortex [41,42,43], which evaluates how the correlation between the activities of neurons influences the synaptic weight of the connection between them. We find that after the self-organization of the network, a large fraction of the total synaptic weight in the network is distributed to a small fraction of connections between neurons with the strongest correlation (Figure 3D, fraction of connections for 50% of the total synaptic weight indicated in title). This finding is in line with experimental results from visual cortex [41,42,43] indicating that the here discussed self-organization of activity and connectivity is biologically plausible.

### 3.6. Is the Spread-Out of Stimulus Representation Energy-Efficient?

One reason why the long-range connectivity between layers is sparse may be to save energy. A cost-efficient implementation of neural computation provides an evolutionary advantage. Hence, it is assumed that networks are arranged such that the total wiring cost stays small [44,45]. However, to allow for a proper readout with less long-range connections (LRC), our network uses strong recurrent or short-range connections (SRC). Thus, it is unclear whether and under which conditions the self-organization principles discovered here decrease the metabolic cost of the network structure.

To investigate this, we assume that the metabolic cost *E* to maintain the connectivity of the network is proportional to a weighted sum of number of short-range connections $n_{SRC}$ and the number of long-range connections $n_{LRC}$: $E\propto E_{LRC}n_{LRC}+E_{SRC}n_{SRC}$, where $E_{LRC}$ and $E_{SRC}$ signify the metabolic cost per long-range and short-range connection, respectively. Using the ratio $\gamma =E_{LRC}/E_{SRC}$, which indicates how much more expensive a long-range connection is compared to a short-range connections, this can be simplified to

$$E\propto \gamma n_{LRC}+n_{SRC}.$$

Hereby, both $n_{SRC}$ and $n_{LRC}$ are determined by the architecture of the network. In the following we will analyze the metabolic cost for the class of network architectures which emerge during the above studied self-organization process. In these networks, a fraction *f* of the neurons are assumed to acquire a tuning and to respond to one stimulus (with $p_{on}=1$), whereas the remaining fraction 1−f of neurons shows no stimulus specific response.

We further assume that, as in our self-organized network, each tuned neuron receives strong short-range connections from all input neurons that are stimulated by its preferred stimulus (if these connections physically exist). Thus, for a network with *N* neurons, the *number of short range connections* is approximately $n_{SRC}=fNp_{con}\beta$, where $p_{con}$ is the connection probability within the network and β the number of stimulated cells for each individual stimulus.

**Figure 4:**
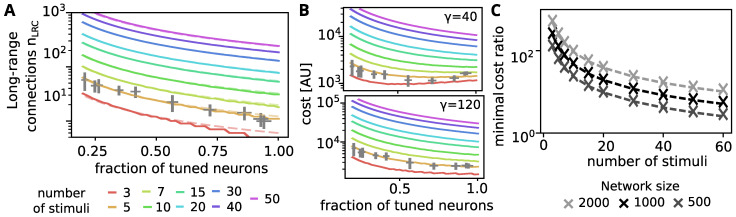
Energy-efficiency of information transmission depending on the fraction of tuned neurons. (**A**) Number of long-range connections needed to sample at least $n_{stim}-1$ different tunings, assuming that a fraction *f* of the neurons are tuned to a single stimulus and all tunings are equally probable. Color indicates the number of stimuli $n_{stim}$. For each number of stimuli, the dependency on *f* is well fit by the function L/f (dashed lines). Grey error-bars mark the simulation corresponding to $n_{stim}=5$ (fraction of single tuned neurons vs long-range connections needed for classification). (**B**) Metabolic cost for connection maintenance assuming that long-range connections are 40× (top) or 120× (bottom) more expensive than the short-range connections in the recurrent network. Grey bars mark simulation results (as above, number of short-range connections from weights above 5nS). (**C**) Maximal cost-ratio between long- and short-range connections for which a number of stimuli (x-axis) is transmitted most cost-efficient through the here presented self-organizing recurrent network. Gray scale indicates the size of the recurrent network, which determines the number of short range connections.

The *number of long-range connections* that are needed to correctly read out the stimulus identity has been determined numerically for different fractions of tuned neurons and different numbers of stimuli through simulation (Figure 4A, solid curves). These numbers of long-range connections match well with those needed to correctly read out from our self-organizing network at different training stages (grey crosses, $n_{LRC}$ and *f* determined as in Figure 1C and Figure 2E, respectively). Using an analytical approximation (see Appendix A), we found that it scales with *f* like $n_{LRC}=L\left(n_{stim}\right)/f$ (best fits shown in Figure 4A, dashed lines).

Using the above relations between the fraction of tuned neurons and the numbers of long- and short-range connections, we can determine the metabolic costs for different cost ratios γ (Figure 4B top/bottom).

For small numbers of stimuli and small cost ratios, there is an optimal fraction of tuned neurons below 1 (Figure 4B top for $n_{stim}\in \left\{3,5\right\}$). This optimal fraction of tuned neurons at which the total metabolic cost is minimized can be expressed as

$$f_{min}=\sqrt{\gamma L\left(n_{stim}\right)/\left(Np_{con}\beta \right)}.$$

For all other cases (where $f_{min}>1$) the metabolic cost is monotonically decreasing with *f* (Figure 4B), such that networks with f=1 are the most cost efficient architecture. Thus, in these cases the here investigated self-organization, which drives the network towards f=1 (see above), is constructing a network architecture that transmits the stimulus information with minimal metabolic cost.

The minimal cost ratio, for which this (f=1) is the case, can be determined from the above expression as

$$\gamma_{min}=\underset{\mathrm{in}-\mathrm{degree}\;\mathrm{of}\;\mathrm{tuned}\;\mathrm{cells}}{\underset{︸}{p_{con}\cdot \beta }}\;\cdot \;\underset{\approx \;\mathrm{tuned}\;\mathrm{cells}\;\mathrm{per}\;\mathrm{stimulus}}{\underset{︸}{N/L\left(n_{stim}\right)}}.$$

The cost ratio is higher the more cells are tuned to each stimulus and the more synapses they receive from the cells stimulated by the inputs $\beta p_{con}$. In Figure 4C we show how this minimal cost ratio scales for different network sizes and number of stimuli (using β=40 and $p_{con}=0.04$ as in the simulations).

We would like to point out, that the metabolic cost to maintain a connection depends on the axonal membrane area [46,47,48], which is in turn proportional to the axonal length. The length-ratios between local projections (order of 100 μm) and long-range projections (order of millimeters) can be expected to be around 10–100. As this already exceeds most of the minimal cost ratios determined in Figure 4C, the here discussed self-organization, which entails that every neuron in the network responds to one stimulus, may indeed be a biologically plausible mechanism to ensure a metabolically cost efficient routing of stimulus information.

## 4. Discussion

In this study we used a well established model of the self-organization of cortical connectivity and demonstrated that the resulting intra-layer connectivity supports information processing and transmission using sparse long-range connections to other brain areas higher or deeper in the cortical hierarchy. Specifically, we demonstrated that the stimulated neurons form feed-forward projections into the unstimulated parts of the network and thereby acquire more neurons to represent the stimulus by rapid stereotypical spiking responses. Hereby, each neuron only receives feed-forward projections from one externally stimulated group such that the network is partitioned into parts with different preferred stimuli (Figure 3F). Finally we showed that the self-organization is consistent with experimental findings and leads to metabolically cost efficient network architectures.

### 4.1. Model Predictions

Several pre- and postdictions can be derived from our model: First of all, it predicts that each neuron is tuned to a stimulus. This is in line with neurons establishing new tunings when they are deafferentiated (e.g., [49]). Note, however, in a given experiment it is still possible to observe neurons which are not tuned to any experimental parameter as the animals still encounter other stimuli outside of the experiment.

Secondly, we see that an increasing training and adaptation time increases the response probabilities of the neurons. Hence, the neural responses to a stimulus will become more and more stereotypical when training continues. Similar effects have been observed in the olfactory domain [50].

Most importantly, however, we predict that an increased entrainment to a set of stimuli will enable the identification of the stimulus from smaller and smaller subsets of neurons. This might be verified by future experiments where learning is tracked in animals with implanted electrode arrays.

### 4.2. Limitations and Possible Extensions

In this study we focused on routing of uncorrelated stimuli which are moreover conveyed to disjunct or orthogonal groups of neurons. Interestingly, the sets of neurons responding to the stimuli are also disjunct/orthogonal such that this assumption is self-consistent with the transmission of stimulus information over many layers. However, often there is a significant correlation between presented stimuli and the corresponding responses. This may lead to cells with mixed responses that support complex computations [51]. Moreover, stimuli might not stem from distinct classes but rather from a continuum, as for example orientation and speed of bars and moving gratings in the visual system. In this case we would also expect more continuous tuning curves. An investigation of the self-organization and information routing in response to correlated stimuli is therefore an interesting direction for future work.

## Figures and Tables

**Table 1:** Simulation parameter values.

Parameter	Value	Parameter	Value
$g_{\mathrm{leak}}$	30 nS	$v_{\mathrm{rest}}$	−70 mV
$c_{\mathrm{membrane}}$	300 pF	$\tau_{\mathrm{membrane}}$	20 ms
$\tau_{\mathrm{ampa}}$	2 ms	$\tau_{\mathrm{gaba}}$	5 ms
$e_{\mathrm{ampa}}$	0 mV	$e_{\mathrm{gaba}}$	−85 mV
$\eta_{\mathrm{decay}}^{\mathrm{ip}}$	0.2 mV/s	$\eta_{\mathrm{spike}}^{\mathrm{ip}}$	0.066 mV
$\sigma_{\mathrm{noise}}$	1 mV	$\tau_{\mathrm{noise}}$	20 ms
$A_{+}$	0.05 nS	$A_{-}$	0.05 nS
$\tau_{+}$	20 ms	$\tau_{-}$	20 ms
		$W_{\mathrm{total}}$	50 nS

## Data Availability

The data presented in this study are available in the article.

## Abbreviations

The following abbreviations are used in this manuscript:

MDPI	Multidisciplinary Digital Publishing Institute
STDP	Spike-timing-dependent plasticity

## Appendix A. Estimation for the Number of Needed Long-Range Connections

Here we approximate the number of long-range connections that is needed to discern $n_{stim}$ different stimuli. We assume that they randomly emerge from cells in the network. Hereby a fraction *f* is tuned to one of the stimuli (equally distributed, whereas a fraction of 1−f is untuned.

A reliable readout needs to sample at least from $n_{stim}-1$ different stimuli. Thus, to determine the minimal number of long-range connection, we then determine the number of samples for which the probability to observe at least $n_{stim}-1$ different tunings is above 95%. Using the probability that two stimuli are not chosen within the first $n_{LRC}$ connections, we get

$$0.95>1-\frac{n_{stim}\left(n_{stim}-1\right)}{2}{\left[1-2\cdot f\cdot \frac{1}{n_{stim}}\right]}^{n_{LRC}}.$$

Hence, using ln(1−x)≈−x, we obtain

$$n_{LRC}>\frac{\ln(2\cdot 0.05/n_{stim}/\left(n_{stim}-1\right))}{\ln\left(1-2\cdot f\cdot \frac{1}{n_{stim}}\right)}\approx \frac{n_{stim}}{2f}\ln\left(\frac{n_{stim}\left(n_{stim}-1\right)}{2\cdot 0.05}\right)=:L\left(n_{stim}\right)/f.$$
